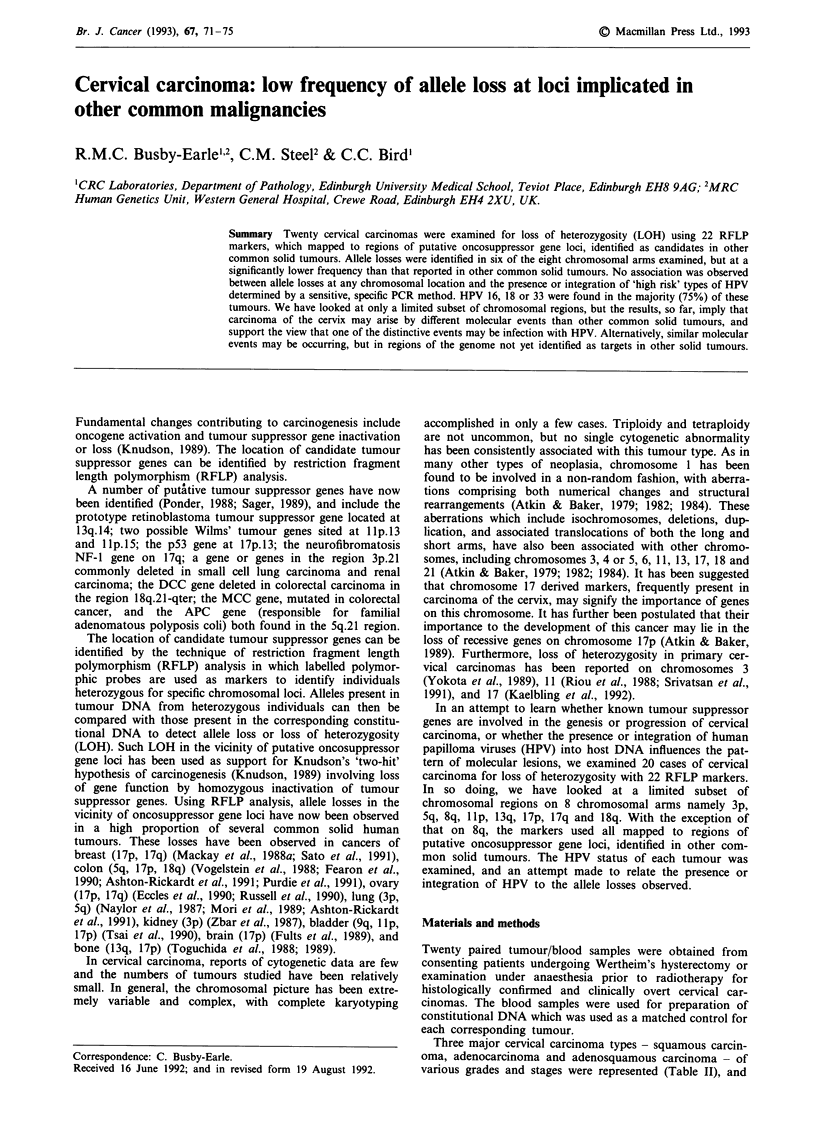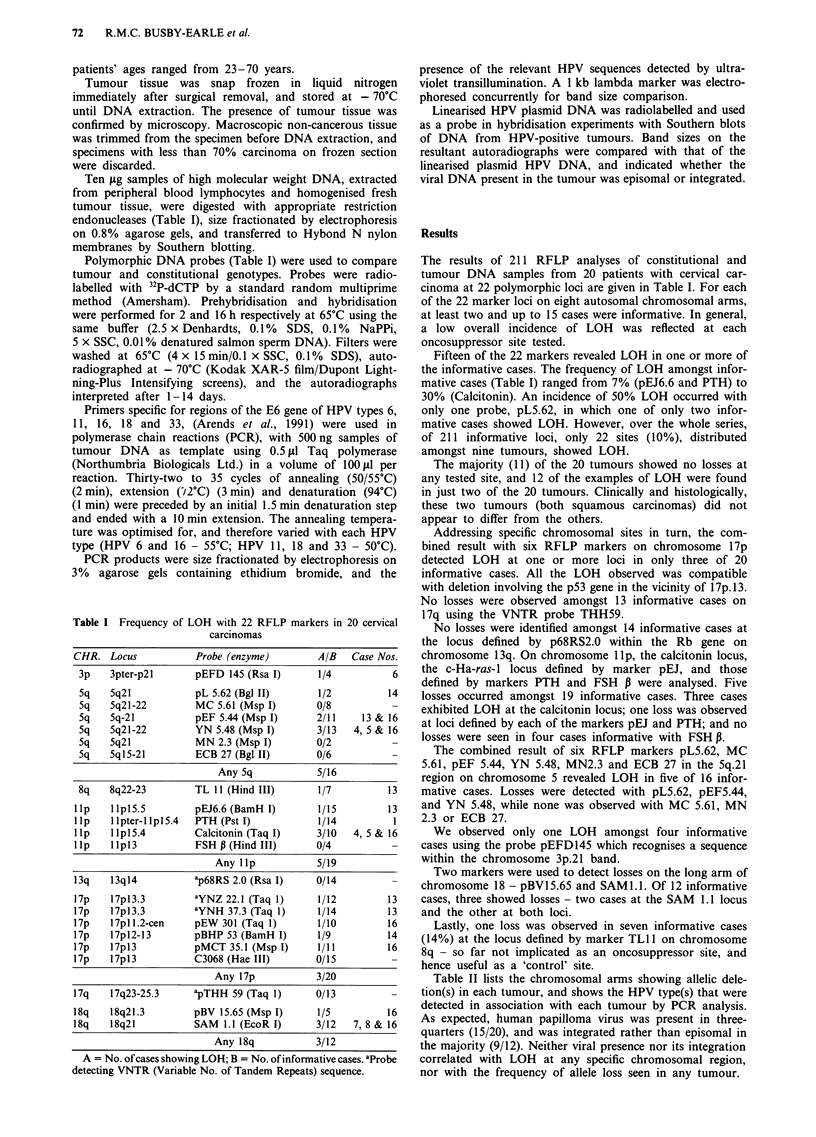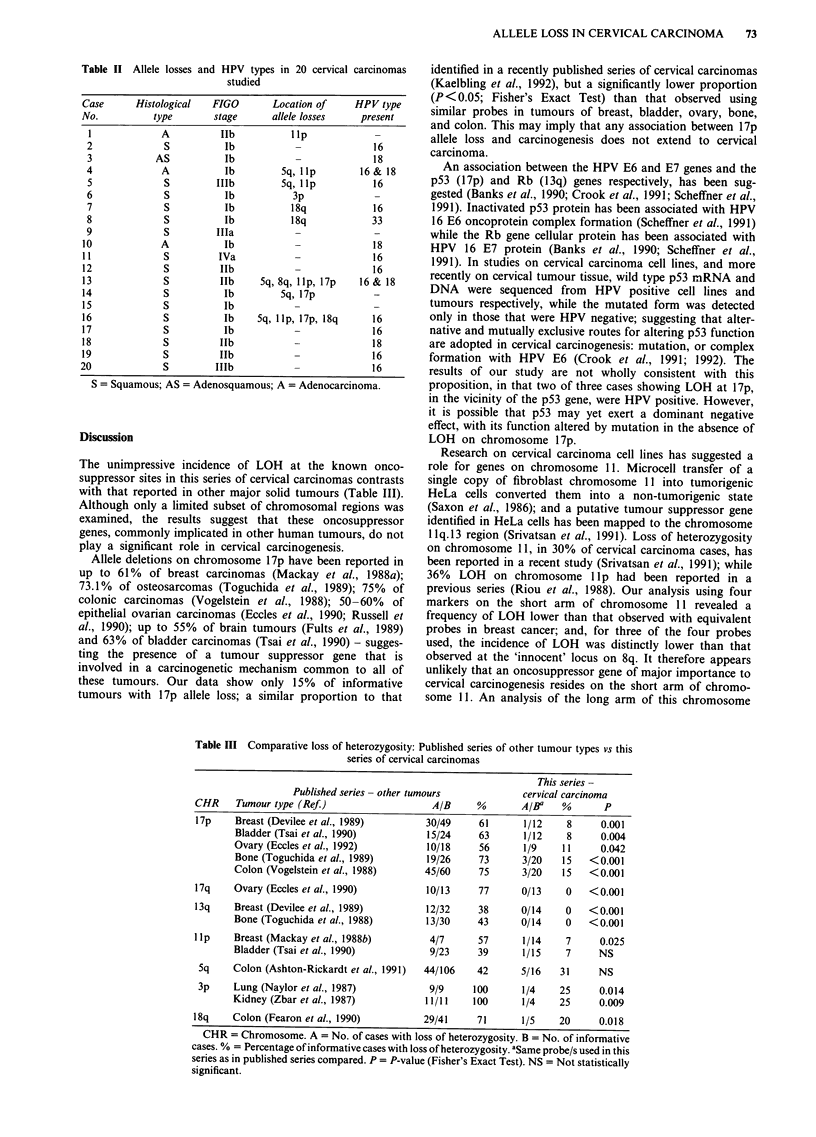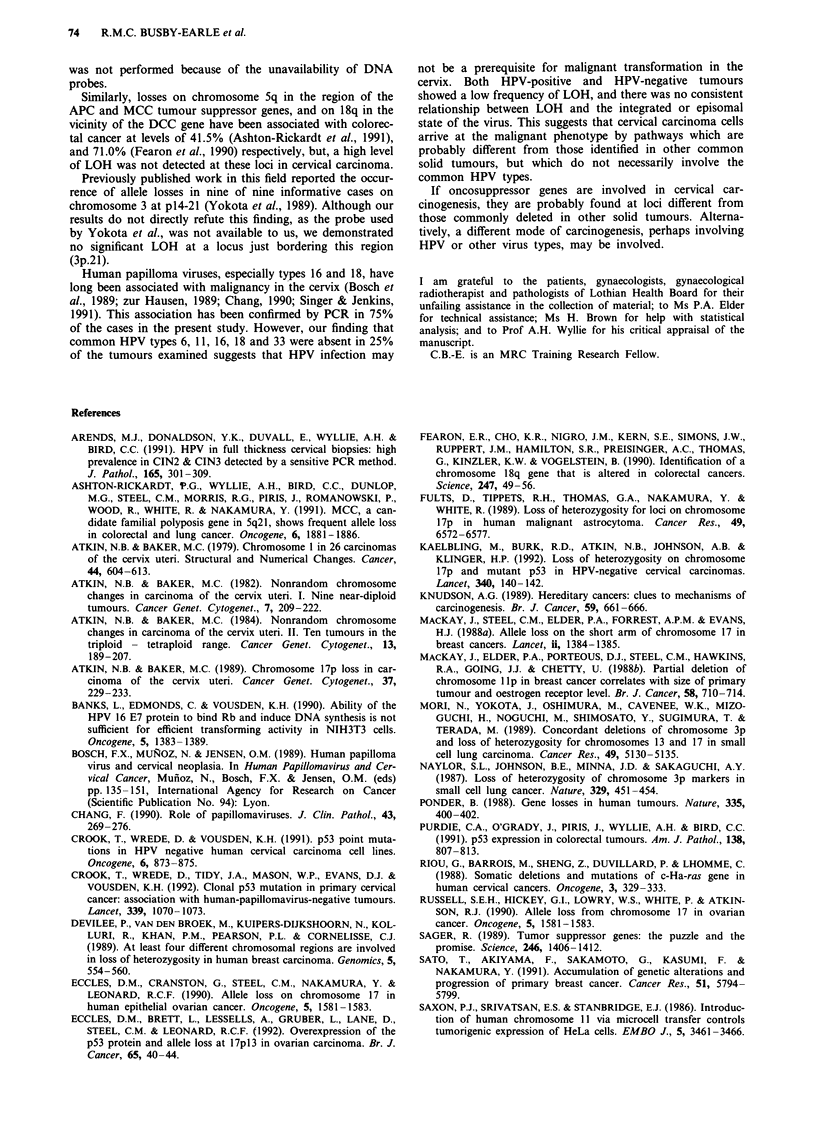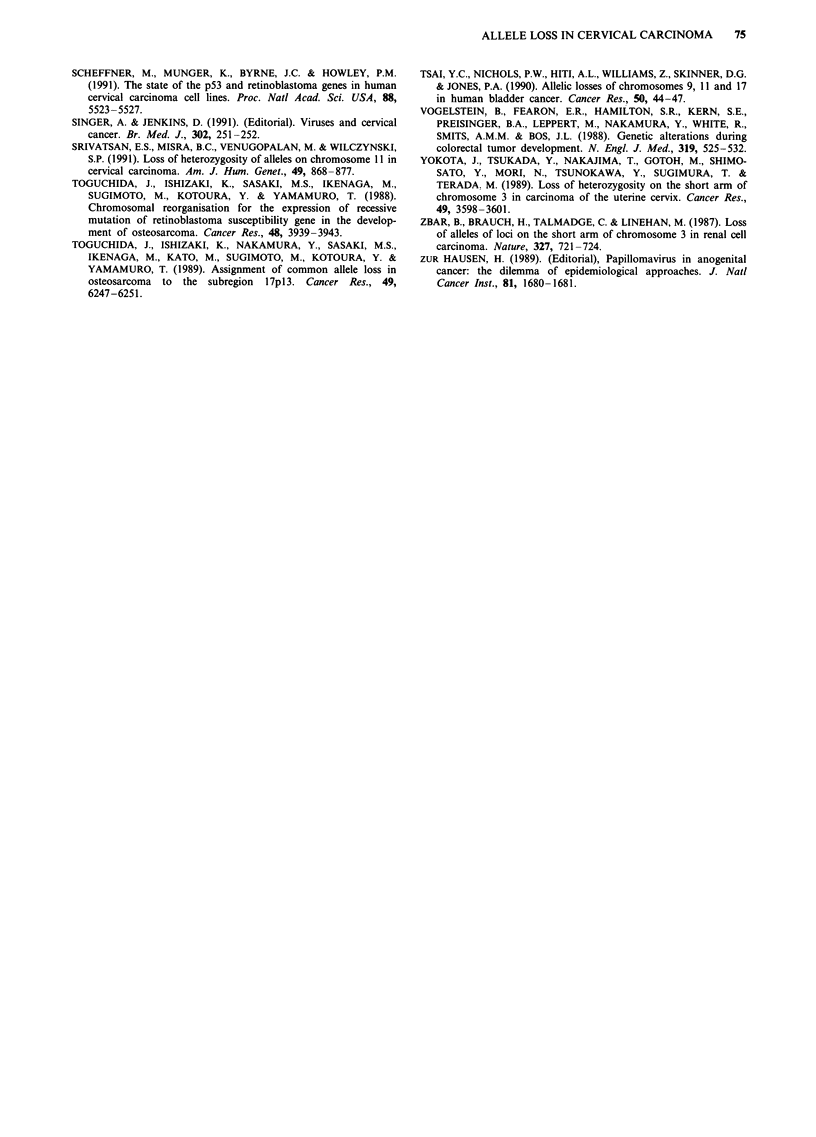# Cervical carcinoma: low frequency of allele loss at loci implicated in other common malignancies.

**DOI:** 10.1038/bjc.1993.11

**Published:** 1993-01

**Authors:** R. M. Busby-Earle, C. M. Steel, C. C. Bird

**Affiliations:** Department of Pathology, Edinburgh University Medical School, UK.

## Abstract

Twenty cervical carcinomas were examined for loss of heterozygosity (LOH) using 22 RFLP markers, which mapped to regions of putative oncosuppressor gene loci, identified as candidates in other common solid tumours. Allele losses were identified in six of the eight chromosomal arms examined, but at a significantly lower frequency than that reported in other common solid tumours. No association was observed between allele losses at any chromosomal location and the presence or integration of 'high risk' types of HPV determined by a sensitive, specific PCR method. HPV 16, 18 or 33 were found in the majority (75%) of these tumours. We have looked at only a limited subset of chromosomal regions, but the results, so far, imply that carcinoma of the cervix may arise by different molecular events than other common solid tumours, and support the view that one of the distinctive events may be infection with HPV. Alternatively, similar molecular events may be occurring, but in regions of the genome not yet identified as targets in other solid tumours.


Br. J. Cancer (1993), 67, 71-75 © Macmillan Press Ltd., 1993

### Cervical carcinoma: low frequency of allele loss at loci implicated in other common malignancies

R.M.C. Busby-Earle[1,2], C.M. Steel[2] & C.C. Bird[1]

[1]*CRC Laboratories, Department of Pathology, Edinburgh University Medical School, Teviot Place, Edinburgh EH8 9AG;* [2]*MRC Human Genetics Unit, Western General Hospital, Crewe Road, Edinburgh EH4 2XU, UK.*

**Summary** Twenty cervical carcinomas were examined for loss of heterozygosity (LOH) using 22 RFLP markers, which mapped to regions of putative oncosuppressor gene loci, identified as candidates in other common solid tumours. Allele losses were identified in six of the eight chromosomal arms examined, but at a significantly lower frequency than that reported in other common solid tumours. No association was observed between allele losses at any chromosomal location and the presence or integration of 'high risk' types of HPV determined by a sensitive, specific PCR method. HPV 16, 18 or 33 were found in the majority (75%) of these tumours. We have looked at only a limited subset of chromosomal regions, but the results, so far, imply that carcinoma of the cervix may arise by different molecular events than other common solid tumours, and support the view that one of the distinctive events may be infection with HPV. Alternatively, similar molecular events may be occurring, but in regions of the genome not yet identified as targets in other solid tumours.

Fundamental changes contributing to carcinogenesis include oncogene activation and tumour suppressor gene inactivation or loss (Knudson, 1989). The location of candidate tumour suppressor genes can be identified by restriction fragment length polymorphism (RFLP) analysis.

A number of putative tumour suppressor genes have now been identified (Ponder, 1988; Sager, 1989), and include the prototype retinoblastoma tumour suppressor gene located at 13q.14; two possible Wilms' tumour genes sited at 11p.13 and 11p.15; the p53 gene at 17p.13; the neurofibromatosis NF-1 gene on 17q; a gene or genes in the region 3p.21 commonly deleted in small cell lung carcinoma and renal carcinoma; the DCC gene deleted in colorectal carcinoma in the region 18q.21-qter; the MCC gene, mutated in colorectal cancer, and the APC gene (responsible for familial adenomatous polyposis coli) both found in the 5q.21 region.

The location of candidate tumour suppressor genes can be identified by the technique of restriction fragment length polymorphism (RFLP) analysis in which labelled polymorphic probes are used as markers to identify individuals heterozygous for specific chromosomal loci. Alleles present in tumour DNA from heterozygous individuals can then be compared with those present in the corresponding constitutional DNA to detect allele loss or loss of heterozygosity (LOH). Such LOH in the vicinity of putative oncosuppressor gene loci has been used as support for Knudson's 'two-hit' hypothesis of carcinogenesis (Knudson, 1989) involving loss of gene function by homozygous inactivation of tumour suppressor genes. Using RFLP analysis, allele losses in the vicinity of oncosuppressor gene loci have now been observed in a high proportion of several common solid human tumours. These losses have been observed in cancers of breast (17p, 17q) (Mackay *et al.*, 1988*a*; Sato *et al.*, 1991), colon (5q, 17p, 18q) (Vogelstein *et al.*, 1988; Fearon *et al.*, 1990; Ashton-Rickardt *et al.*, 1991; Purdie *et al.*, 1991), ovary (17p, 17q) (Eccles *et al.*, 1990; Russell *et al.*, 1990), lung (3p, 5q) (Naylor *et al.*, 1987; Mori *et al.*, 1989; Ashton-Rickardt *et al.*, 1991), kidney (3p) (Zbar *et al.*, 1987), bladder (9q, 11p, 17p) (Tsai *et al.*, 1990), brain (17p) (Fults *et al.*, 1989), and bone (13q, 17p) (Toguchida *et al.*, 1988; 1989).

In cervical carcinoma, reports of cytogenetic data are few and the numbers of tumours studied have been relatively small. In general, the chromosomal picture has been extremely variable and complex, with complete karyotyping accomplished in only a few cases. Triploidy and tetraploidy are not uncommon, but no single cytogenetic abnormality has been consistently associated with this tumour type. As in many other types of neoplasia, chromosome 1 has been found to be involved in a non-random fashion, with aberrations comprising both numerical changes and structural rearrangements (Atkin & Baker, 1979; 1982; 1984). These aberrations which include isochromosomes, deletions, duplication, and associated translocations of both the long and short arms, have also been associated with other chromosomes, including chromosomes 3, 4 or 5, 6, 11, 13, 17, 18 and 21 (Atkin & Baker, 1979; 1982; 1984). It has been suggested that chromosome 17 derived markers, frequently present in carcinoma of the cervix, may signify the importance of genes on this chromosome. It has further been postulated that their importance to the development of this cancer may lie in the loss of recessive genes on chromosome 17p (Atkin & Baker, 1989). Furthermore, loss of heterozygosity in primary cervical carcinomas has been reported on chromosomes 3 (Yokota *et al.*, 1989), 11 (Riou *et al.*, 1988; Srivatsan *et al.*, 1991), and 17 (Kaelbling *et al.*, 1992).

In an attempt to learn whether known tumour suppressor genes are involved in the genesis or progression of cervical carcinoma, or whether the presence or integration of human papilloma viruses (HPV) into host DNA influences the pattern of molecular lesions, we examined 20 cases of cervical carcinoma for loss of heterozygosity with 22 RFLP markers. In so doing, we have looked at a limited subset of chromosomal regions on 8 chromosomal arms namely 3p, 5q, 8q, 11p, 13q, 17p, 17q and 18q. With the exception of that on 8q, the markers used all mapped to regions of putative oncosuppressor gene loci, identified in other common solid tumours. The HPV status of each tumour was examined, and an attempt made to relate the presence or integration of HPV to the allele losses observed.

### Materials and methods

Twenty paired tumour/blood samples were obtained from consenting patients undergoing Wertheim's hysterectomy or examination under anaesthesia prior to radiotherapy for histologically confirmed and clinically overt cervical carcinomas. The blood samples were used for preparation of constitutional DNA which was used as a matched control for each corresponding tumour.

Three major cervical carcinoma types – squamous carcinoma, adenocarcinoma and adenosquamous carcinoma – of various grades and stages were represented (Table II), and

Correspondence: C. Busby-Earle.
Received 16 June 1992; and in revised form 19 August 1992.

patients' ages ranged from 23–70 years.

Tumour tissue was snap frozen in liquid nitrogen immediately after surgical removal, and stored at – 70°C until DNA extraction. The presence of tumour tissue was confirmed by microscopy. Macroscopic non-cancerous tissue was trimmed from the specimen before DNA extraction, and specimens with less than 70% carcinoma on frozen section were discarded.

Ten μg samples of high molecular weight DNA, extracted from peripheral blood lymphocytes and homogenised fresh tumour tissue, were digested with appropriate restriction endonucleases (Table I), size fractionated by electrophoresis on 0.8% agarose gels, and transferred to Hybond N nylon membranes by Southern blotting.

Polymorphic DNA probes (Table I) were used to compare tumour and constitutional genotypes. Probes were radiolabelled with $^{32}$P-dCTP by a standard random multiprime method (Amersham). Prehybridisation and hybridisation were performed for 2 and 16 h respectively at 65°C using the same buffer (2.5 × Denhardts, 0.1% SDS, 0.1% NaPPi, 5 × SSC, 0.01% denatured salmon sperm DNA). Filters were washed at 65°C (4 × 15 min/0.1 × SSC, 0.1% SDS), autoradiographed at – 70°C (Kodak XAR-5 film/Dupont Lightning-Plus Intensifying screens), and the autoradiographs interpreted after 1–14 days.

Primers specific for regions of the E6 gene of HPV types 6, 11, 16, 18 and 33, (Arends *et al.*, 1991) were used in polymerase chain reactions (PCR), with 500 ng samples of tumour DNA as template using 0.5 μl Taq polymerase (Northumbria Biologicals Ltd.) in a volume of 100 μl per reaction. Thirty-two to 35 cycles of annealing (50/55°C) (2 min), extension (72°C) (3 min) and denaturation (94°C) (1 min) were preceded by an initial 1.5 min denaturation step and ended with a 10 min extension. The annealing temperature was optimised for, and therefore varied with each HPV type (HPV 6 and 16 – 55°C; HPV 11, 18 and 33 – 50°C).

PCR products were size fractionated by electrophoresis on 3% agarose gels containing ethidium bromide, and the presence of the relevant HPV sequences detected by ultraviolet transillumination. A 1 kb lambda marker was electrophoresed concurrently for band size comparison.

Linearised HPV plasmid DNA was radiolabelled and used as a probe in hybridisation experiments with Southern blots of DNA from HPV-positive tumours. Band sizes on the resultant autoradiographs were compared with that of the linearised plasmid HPV DNA, and indicated whether the viral DNA present in the tumour was episomal or integrated.

**Table I** Frequency of LOH with 22 RFLP markers in 20 cervical carcinomas

| *CHR.* | *Locus* | *Probe (enzyme)* | *A/B* | *Case Nos.* |
|---|---|---|---|---|
| 3p | 3pter-p21 | pEFD 145 (Rsa I) | 1/4 | 6 |
| 5q | 5q21 | pL 5.62 (Bgl II) | 1/2 | 14 |
| 5q | 5q21-22 | MC 5.61 (Msp I) | 0/8 | – |
| 5q | 5q-21 | pEF 5.44 (Msp I) | 2/11 | 13 & 16 |
| 5q | 5q21-22 | YN 5.48 (Msp I) | 3/13 | 4, 5 & 16 |
| 5q | 5q21 | MN 2.3 (Msp I) | 0/2 | – |
| 5q | 5q15-21 | ECB 27 (Bgl II) | 0/6 | – |
| | | Any 5q | 5/16 | |
| 8q | 8q22-23 | TL 11 (Hind III) | 1/7 | 13 |
| 11p | 11p15.5 | pEJ6.6 (BamH I) | 1/15 | 13 |
| 11p | 11pter-11p15.4 | PTH (Pst I) | 1/14 | 1 |
| 11p | 11p15.4 | Calcitonin (Taq I) | 3/10 | 4, 5 & 16 |
| 11p | 11p13 | FSH β (Hind III) | 0/4 | – |
| | | Any 11p | 5/19 | |
| 13q | 13q14 | [a]p68RS 2.0 (Rsa I) | 0/14 | – |
| 17p | 17p13.3 | [a]YNZ 22.1 (Taq 1) | 1/12 | 13 |
| 17p | 17p13.3 | [a]YNH 37.3 (Taq 1) | 1/14 | 13 |
| 17p | 17p11.2-cen | pEW 301 (Taq 1) | 1/10 | 16 |
| 17p | 17p12-13 | pBHP 53 (BamH I) | 1/9 | 14 |
| 17p | 17p13 | pMCT 35.1 (Msp I) | 1/11 | 16 |
| 17p | 17p13 | C3068 (Hae III) | 0/15 | – |
| | | Any 17p | 3/20 | |
| 17q | 17q23-25.3 | [a]pTHH 59 (Taq 1) | 0/13 | – |
| 18q | 18q21.3 | pBV 15.65 (Msp I) | 1/5 | 16 |
| 18q | 18q21 | SAM 1.1 (EcoR I) | 3/12 | 7, 8 & 16 |
| | | Any 18q | 3/12 | |

A = No. of cases showing LOH; B = No. of informative cases. [a]Probe detecting VNTR (Variable No. of Tandem Repeats) sequence.

### Results

The results of 211 RFLP analyses of constitutional and tumour DNA samples from 20 patients with cervical carcinoma at 22 polymorphic loci are given in Table I. For each of the 22 marker loci on eight autosomal chromosomal arms, at least two and up to 15 cases were informative. In general, a low overall incidence of LOH was reflected at each oncosuppressor site tested.

Fifteen of the 22 markers revealed LOH in one or more of the informative cases. The frequency of LOH amongst informative cases (Table I) ranged from 7% (pEJ6.6 and PTH) to 30% (Calcitonin). An incidence of 50% LOH occurred with only one probe, pL5.62, in which one of only two informative cases showed LOH. However, over the whole series, of 211 informative loci, only 22 sites (10%), distributed amongst nine tumours, showed LOH.

The majority (11) of the 20 tumours showed no losses at any tested site, and 12 of the examples of LOH were found in just two of the 20 tumours. Clinically and histologically, these two tumours (both squamous carcinomas) did not appear to differ from the others.

Addressing specific chromosomal sites in turn, the combined result with six RFLP markers on chromosome 17p detected LOH at one or more loci in only three of 20 informative cases. All the LOH observed was compatible with deletion involving the p53 gene in the vicinity of 17p.13. No losses were observed amongst 13 informative cases on 17q using the VNTR probe THH59.

No losses were identified amongst 14 informative cases at the locus defined by p68RS2.0 within the Rb gene on chromosome 13q. On chromosome 11p, the calcitonin locus, the c-Ha-*ras*-1 locus defined by marker pEJ, and those defined by markers PTH and FSH β were analysed. Five losses occurred amongst 19 informative cases. Three cases exhibited LOH at the calcitonin locus; one loss was observed at loci defined by each of the markers pEJ and PTH; and no losses were seen in four cases informative with FSH β.

The combined result of six RFLP markers pL5.62, MC 5.61, pEF 5.44, YN 5.48, MN2.3 and ECB 27 in the 5q.21 region on chromosome 5 revealed LOH in five of 16 informative cases. Losses were detected with pL5.62, pEF5.44, and YN 5.48, while none was observed with MC 5.61, MN 2.3 or ECB 27.

We observed only one LOH amongst four informative cases using the probe pEFD145 which recognises a sequence within the chromosome 3p.21 band.

Two markers were used to detect losses on the long arm of chromosome 18 – pBV15.65 and SAM1.1. Of 12 informative cases, three showed losses – two cases at the SAM 1.1 locus and the other at both loci.

Lastly, one loss was observed in seven informative cases (14%) at the locus defined by marker TL11 on chromosome 8q – so far not implicated as an oncosuppressor site, and hence useful as a 'control' site.

Table II lists the chromosomal arms showing allelic deletion(s) in each tumour, and shows the HPV type(s) that were detected in association with each tumour by PCR analysis. As expected, human papilloma virus was present in three-quarters (15/20), and was integrated rather than episomal in the majority (9/12). Neither viral presence nor its integration correlated with LOH at any specific chromosomal region, nor with the frequency of allele loss seen in any tumour.

**Table II** Allele losses and HPV types in 20 cervical carcinomas studied

| *Case No.* | *Histological type* | *FIGO stage* | *Location of allele losses* | *HPV type present* |
|---|---|---|---|---|
| 1 | A | IIb | 11p | – |
| 2 | S | Ib | – | 16 |
| 3 | AS | Ib | – | 18 |
| 4 | A | Ib | 5q, 11p | 16 & 18 |
| 5 | S | IIIb | 5q, 11p | 16 |
| 6 | S | Ib | 3p | – |
| 7 | S | Ib | 18q | 16 |
| 8 | S | Ib | 18q | 33 |
| 9 | S | IIIa | – | – |
| 10 | A | Ib | – | 18 |
| 11 | S | IVa | – | 16 |
| 12 | S | IIb | – | 16 |
| 13 | S | IIb | 5q, 8q, 11p, 17p | 16 & 18 |
| 14 | S | Ib | 5q, 17p | – |
| 15 | S | Ib | – | – |
| 16 | S | Ib | 5q, 11p, 17p, 18q | 16 |
| 17 | S | Ib | – | 16 |
| 18 | S | IIb | – | 18 |
| 19 | S | IIb | – | 16 |
| 20 | S | IIIb | – | 16 |

S = Squamous; AS = Adenosquamous; A = Adenocarcinoma.

### Discussion

The unimpressive incidence of LOH at the known oncosuppressor sites in this series of cervical carcinomas contrasts with that reported in other major solid tumours (Table III). Although only a limited subset of chromosomal regions was examined, the results suggest that these oncosuppressor genes, commonly implicated in other human tumours, do not play a significant role in cervical carcinogenesis.

Allele deletions on chromosome 17p have been reported in up to 61% of breast carcinomas (Mackay *et al.*, 1988*a*); 73.1% of osteosarcomas (Toguchida *et al.*, 1989); 75% of colonic carcinomas (Vogelstein *et al.*, 1988); 50–60% of epithelial ovarian carcinomas (Eccles *et al.*, 1990; Russell *et al.*, 1990); up to 55% of brain tumours (Fults *et al.*, 1989) and 63% of bladder carcinomas (Tsai *et al.*, 1990) – suggesting the presence of a tumour suppressor gene that is involved in a carcinogenetic mechanism common to all of these tumours. Our data show only 15% of informative tumours with 17p allele loss; a similar proportion to that identified in a recently published series of cervical carcinomas (Kaelbling *et al.*, 1992), but a significantly lower proportion ($P<0.05$; Fisher's Exact Test) than that observed using similar probes in tumours of breast, bladder, ovary, bone, and colon. This may imply that any association between 17p allele loss and carcinogenesis does not extend to cervical carcinoma.

An association between the HPV E6 and E7 genes and the p53 (17p) and Rb (13q) genes respectively, has been suggested (Banks *et al.*, 1990; Crook *et al.*, 1991; Scheffner *et al.*, 1991). Inactivated p53 protein has been associated with HPV 16 E6 oncoprotein complex formation (Scheffner *et al.*, 1991) while the Rb gene cellular protein has been associated with HPV 16 E7 protein (Banks *et al.*, 1990; Scheffner *et al.*, 1991). In studies on cervical carcinoma cell lines, and more recently on cervical tumour tissue, wild type p53 mRNA and DNA were sequenced from HPV positive cell lines and tumours respectively, while the mutated form was detected only in those that were HPV negative; suggesting that alternative and mutually exclusive routes for altering p53 function are adopted in cervical carcinogenesis: mutation, or complex formation with HPV E6 (Crook *et al.*, 1991; 1992). The results of our study are not wholly consistent with this proposition, in that two of three cases showing LOH at 17p, in the vicinity of the p53 gene, were HPV positive. However, it is possible that p53 may yet exert a dominant negative effect, with its function altered by mutation in the absence of LOH on chromosome 17p.

Research on cervical carcinoma cell lines has suggested a role for genes on chromosome 11. Microcell transfer of a single copy of fibroblast chromosome 11 into tumorigenic HeLa cells converted them into a non-tumorigenic state (Saxon *et al.*, 1986); and a putative tumour suppressor gene identified in HeLa cells has been mapped to the chromosome 11q.13 region (Srivatsan *et al.*, 1991). Loss of heterozygosity on chromosome 11, in 30% of cervical carcinoma cases, has been reported in a recent study (Srivatsan *et al.*, 1991); while 36% LOH on chromosome 11p had been reported in a previous series (Riou *et al.*, 1988). Our analysis using four markers on the short arm of chromosome 11 revealed a frequency of LOH lower than that observed with equivalent probes in breast cancer; and, for three of the four probes used, the incidence of LOH was distinctly lower than that observed at the 'innocent' locus on 8q. It therefore appears unlikely that an oncosuppressor gene of major importance to cervical carcinogenesis resides on the short arm of chromosome 11. An analysis of the long arm of this chromosome

**Table III** Comparative loss of heterozygosity: Published series of other tumour types *vs* this series of cervical carcinomas

| | *Published series – other tumours* | | | *This series – cervical carcinoma* | | |
|---|---|---|---|---|---|---|
| *CHR* | *Tumour type (Ref.)* | *A/B* | *%* | *A/B*[a] | *%* | *P* |
| 17p | Breast (Devilee *et al.*, 1989) | 30/49 | 61 | 1/12 | 8 | 0.001 |
| | Bladder (Tsai *et al.*, 1990) | 15/24 | 63 | 1/12 | 8 | 0.004 |
| | Ovary (Eccles *et al.*, 1992) | 10/18 | 56 | 1/9 | 11 | 0.042 |
| | Bone (Toguchida *et al.*, 1989) | 19/26 | 73 | 3/20 | 15 | <0.001 |
| | Colon (Vogelstein *et al.*, 1988) | 45/60 | 75 | 3/20 | 15 | <0.001 |
| 17q | Ovary (Eccles *et al.*, 1990) | 10/13 | 77 | 0/13 | 0 | <0.001 |
| 13q | Breast (Devilee *et al.*, 1989) | 12/32 | 38 | 0/14 | 0 | <0.001 |
| | Bone (Toguchida *et al.*, 1988) | 13/30 | 43 | 0/14 | 0 | <0.001 |
| 11p | Breast (Mackay *et al.*, 1988*b*) | 4/7 | 57 | 1/14 | 7 | 0.025 |
| | Bladder (Tsai *et al.*, 1990) | 9/23 | 39 | 1/15 | 7 | NS |
| 5q | Colon (Ashton-Rickardt *et al.*, 1991) | 44/106 | 42 | 5/16 | 31 | NS |
| 3p | Lung (Naylor *et al.*, 1987) | 9/9 | 100 | 1/4 | 25 | 0.014 |
| | Kidney (Zbar *et al.*, 1987) | 11/11 | 100 | 1/4 | 25 | 0.009 |
| 18q | Colon (Fearon *et al.*, 1990) | 29/41 | 71 | 1/5 | 20 | 0.018 |

CHR = Chromosome. A = No. of cases with loss of heterozygosity. B = No. of informative cases. % = Percentage of informative cases with loss of heterozygosity. [a]Same probe/s used in this series as in published series compared. P = P-value (Fisher's Exact Test). NS = Not statistically significant.

was not performed because of the unavailability of DNA probes.

Similarly, losses on chromosome 5q in the region of the APC and MCC tumour suppressor genes, and on 18q in the vicinity of the DCC gene have been associated with colorectal cancer at levels of 41.5% (Ashton-Rickardt *et al.*, 1991), and 71.0% (Fearon *et al.*, 1990) respectively, but, a high level of LOH was not detected at these loci in cervical carcinoma.

Previously published work in this field reported the occurrence of allele losses in nine of nine informative cases on chromosome 3 at p14-21 (Yokota *et al.*, 1989). Although our results do not directly refute this finding, as the probe used by Yokota *et al.*, was not available to us, we demonstrated no significant LOH at a locus just bordering this region (3p.21).

Human papilloma viruses, especially types 16 and 18, have long been associated with malignancy in the cervix (Bosch *et al.*, 1989; zur Hausen, 1989; Chang, 1990; Singer & Jenkins, 1991). This association has been confirmed by PCR in 75% of the cases in the present study. However, our finding that common HPV types 6, 11, 16, 18 and 33 were absent in 25% of the tumours examined suggests that HPV infection may not be a prerequisite for malignant transformation in the cervix. Both HPV-positive and HPV-negative tumours showed a low frequency of LOH, and there was no consistent relationship between LOH and the integrated or episomal state of the virus. This suggests that cervical carcinoma cells arrive at the malignant phenotype by pathways which are probably different from those identified in other common solid tumours, but which do not necessarily involve the common HPV types.

If oncosuppressor genes are involved in cervical carcinogenesis, they are probably found at loci different from those commonly deleted in other solid tumours. Alternatively, a different mode of carcinogenesis, perhaps involving HPV or other virus types, may be involved.

I am grateful to the patients, gynaecologists, gynaecological radiotherapist and pathologists of Lothian Health Board for their unfailing assistance in the collection of material; to Ms P.A. Elder for technical assistance; Ms H. Brown for help with statistical analysis; and to Prof A.H. Wyllie for his critical appraisal of the manuscript.

C.B.-E. is an MRC Training Research Fellow.

### References

ARENDS, M.J., DONALDSON, Y.K., DUVALL, E., WYLLIE, A.H. & BIRD, C.C. (1991). HPV in full thickness cervical biopsies: high prevalence in CIN2 & CIN3 detected by a sensitive PCR method. *J. Pathol.*, **165**, 301-309.

ASHTON-RICKARDT, P.G., WYLLIE, A.H., BIRD, C.C., DUNLOP, M.G., STEEL, C.M., MORRIS, R.G., PIRIS, J., ROMANOWSKI, P., WOOD, R., WHITE, R. & NAKAMURA, Y. (1991). MCC, a candidate familial polyposis gene in 5q21, shows frequent allele loss in colorectal and lung cancer. *Oncogene*, **6**, 1881-1886.

ATKIN, N.B. & BAKER, M.C. (1979). Chromosome 1 in 26 carcinomas of the cervix uteri. Structural and Numerical Changes. *Cancer*, **44**, 604-613.

ATKIN, N.B. & BAKER, M.C. (1982). Nonrandom chromosome changes in carcinoma of the cervix uteri. I. Nine near-diploid tumours. *Cancer Genet. Cytogenet.*, **7**, 209-222.

ATKIN, N.B. & BAKER, M.C. (1984). Nonrandom chromosome changes in carcinoma of the cervix uteri. II. Ten tumours in the triploid - tetraploid range. *Cancer Genet. Cytogenet.*, **13**, 189-207.

ATKIN, N.B. & BAKER, M.C. (1989). Chromosome 17p loss in carcinoma of the cervix uteri. *Cancer Genet. Cytogenet.*, **37**, 229-233.

BANKS, L., EDMONDS, C. & VOUSDEN, K.H. (1990). Ability of the HPV 16 E7 protein to bind Rb and induce DNA synthesis is not sufficient for efficient transforming activity in NIH3T3 cells. *Oncogene*, **5**, 1383-1389.

BOSCH, F.X., MUÑOZ, N. & JENSEN, O.M. (1989). Human papilloma virus and cervical neoplasia. In *Human Papillomavirus and Cervical Cancer*, Muñoz, N., Bosch, F.X. & Jensen, O.M. (eds) pp. 135-151, International Agency for Research on Cancer (Scientific Publication No. 94): Lyon.

CHANG, F. (1990). Role of papillomaviruses. *J. Clin. Pathol.*, **43**, 269-276.

CROOK, T., WREDE, D. & VOUSDEN, K.H. (1991). p53 point mutations in HPV negative human cervical carcinoma cell lines. *Oncogene*, **6**, 873-875.

CROOK, T., WREDE, D., TIDY, J.A., MASON, W.P., EVANS, D.J. & VOUSDEN, K.H. (1992). Clonal p53 mutation in primary cervical cancer: association with human-papillomavirus-negative tumours. *Lancet*, **339**, 1070-1073.

DEVILEE, P., VAN DEN BROEK, M., KUIPERS-DIJKSHOORN, N., KOLLURI, R., KHAN, P.M., PEARSON, P.L. & CORNELISSE, C.J. (1989). At least four different chromosomal regions are involved in loss of heterozygosity in human breast carcinoma. *Genomics*, **5**, 554-560.

ECCLES, D.M., CRANSTON, G., STEEL, C.M., NAKAMURA, Y. & LEONARD, R.C.F. (1990). Allele loss on chromosome 17 in human epithelial ovarian cancer. *Oncogene*, **5**, 1581-1583.

ECCLES, D.M., BRETT, L., LESSELLS, A., GRUBER, L., LANE, D., STEEL, C.M. & LEONARD, R.C.F. (1992). Overexpression of the p53 protein and allele loss at 17p13 in ovarian carcinoma. *Br. J. Cancer*, **65**, 40-44.

FEARON, E.R., CHO, K.R., NIGRO, J.M., KERN, S.E., SIMONS, J.W., RUPPERT, J.M., HAMILTON, S.R., PREISINGER, A.C., THOMAS, G., KINZLER, K.W. & VOGELSTEIN, B. (1990). Identification of a chromosome 18q gene that is altered in colorectal cancers. *Science*, **247**, 49-56.

FULTS, D., TIPPETS, R.H., THOMAS, G.A., NAKAMURA, Y. & WHITE, R. (1989). Loss of heterozygosity for loci on chromosome 17p in human malignant astrocytoma. *Cancer Res.*, **49**, 6572-6577.

KAELBLING, M., BURK, R.D., ATKIN, N.B., JOHNSON, A.B. & KLINGER, H.P. (1992). Loss of heterozygosity on chromosome 17p and mutant p53 in HPV-negative cervical carcinomas. *Lancet*, **340**, 140-142.

KNUDSON, A.G. (1989). Hereditary cancers: clues to mechanisms of carcinogenesis. *Br. J. Cancer*, **59**, 661-666.

MACKAY, J., STEEL, C.M., ELDER, P.A., FORREST, A.P.M. & EVANS, H.J. (1988*a*). Allele loss on the short arm of chromosome 17 in breast cancers. *Lancet*, **ii**, 1384-1385.

MACKAY, J., ELDER, P.A., PORTEOUS, D.J., STEEL, C.M., HAWKINS, R.A., GOING, J.J. & CHETTY, U. (1988*b*). Partial deletion of chromosome 11p in breast cancer correlates with size of primary tumour and oestrogen receptor level. *Br. J. Cancer*, **58**, 710-714.

MORI, N., YOKOTA, J., OSHIMURA, M., CAVENEE, W.K., MIZOGUCHI, H., NOGUCHI, M., SHIMOSATO, Y., SUGIMURA, T. & TERADA, M. (1989). Concordant deletions of chromosome 3p and loss of heterozygosity for chromosomes 13 and 17 in small cell lung carcinoma. *Cancer Res.*, **49**, 5130-5135.

NAYLOR, S.L., JOHNSON, B.E., MINNA, J.D. & SAKAGUCHI, A.Y. (1987). Loss of heterozygosity of chromosome 3p markers in small cell lung cancer. *Nature*, **329**, 451-454.

PONDER, B. (1988). Gene losses in human tumours. *Nature*, **335**, 400-402.

PURDIE, C.A., O'GRADY, J., PIRIS, J., WYLLIE, A.H. & BIRD, C.C. (1991). p53 expression in colorectal tumours. *Am. J. Pathol.*, **138**, 807-813.

RIOU, G., BARROIS, M., SHENG, Z., DUVILLARD, P. & LHOMME, C. (1988). Somatic deletions and mutations of c-Ha-*ras* gene in human cervical cancers. *Oncogene*, **3**, 329-333.

RUSSELL, S.E.H., HICKEY, G.I., LOWRY, W.S., WHITE, P. & ATKINSON, R.J. (1990). Allele loss from chromosome 17 in ovarian cancer. *Oncogene*, **5**, 1581-1583.

SAGER, R. (1989). Tumor suppressor genes: the puzzle and the promise. *Science*, **246**, 1406-1412.

SATO, T., AKIYAMA, F., SAKAMOTO, G., KASUMI, F. & NAKAMURA, Y. (1991). Accumulation of genetic alterations and progression of primary breast cancer. *Cancer Res.*, **51**, 5794-5799.

SAXON, P.J., SRIVATSAN, E.S. & STANBRIDGE, E.J. (1986). Introduction of human chromosome 11 via microcell transfer controls tumorigenic expression of HeLa cells. *EMBO J.*, **5**, 3461-3466.

SCHEFFNER, M., MUNGER, K., BYRNE, J.C. & HOWLEY, P.M. (1991). The state of the p53 and retinoblastoma genes in human cervical carcinoma cell lines. *Proc. Natl Acad. Sci. USA*, **88,** 5523–5527.

SINGER, A. & JENKINS, D. (1991). (Editorial). Viruses and cervical cancer. *Br. Med. J.*, **302,** 251–252.

SRIVATSAN, E.S., MISRA, B.C., VENUGOPALAN, M. & WILCZYNSKI, S.P. (1991). Loss of heterozygosity of alleles on chromosome 11 in cervical carcinoma. *Am. J. Hum. Genet.*, **49,** 868–877.

TOGUCHIDA, J., ISHIZAKI, K., SASAKI, M.S., IKENAGA, M., SUGIMOTO, M., KOTOURA, Y. & YAMAMURO, T. (1988). Chromosomal reorganisation for the expression of recessive mutation of retinoblastoma susceptibility gene in the development of osteosarcoma. *Cancer Res.*, **48,** 3939–3943.

TOGUCHIDA, J., ISHIZAKI, K., NAKAMURA, Y., SASAKI, M.S., IKENAGA, M., KATO, M., SUGIMOTO, M., KOTOURA, Y. & YAMAMURO, T. (1989). Assignment of common allele loss in osteosarcoma to the subregion 17p13. *Cancer Res.*, **49,** 6247–6251.

TSAI, Y.C., NICHOLS, P.W., HITI, A.L., WILLIAMS, Z., SKINNER, D.G. & JONES, P.A. (1990). Allelic losses of chromosomes 9, 11 and 17 in human bladder cancer. *Cancer Res.*, **50,** 44–47.

VOGELSTEIN, B., FEARON, E.R., HAMILTON, S.R., KERN, S.E., PREISINGER, B.A., LEPPERT, M., NAKAMURA, Y., WHITE, R., SMITS, A.M.M. & BOS, J.L. (1988). Genetic alterations during colorectal tumor development. *N. Engl. J. Med.*, **319,** 525–532.

YOKOTA, J., TSUKADA, Y., NAKAJIMA, T., GOTOH, M., SHIMOSATO, Y., MORI, N., TSUNOKAWA, Y., SUGIMURA, T. & TERADA, M. (1989). Loss of heterozygosity on the short arm of chromosome 3 in carcinoma of the uterine cervix. *Cancer Res.*, **49,** 3598–3601.

ZBAR, B., BRAUCH, H., TALMADGE, C. & LINEHAN, M. (1987). Loss of alleles of loci on the short arm of chromosome 3 in renal cell carcinoma. *Nature*, **327,** 721–724.

ZUR HAUSEN, H. (1989). (Editorial), Papillomavirus in anogenital cancer: the dilemma of epidemiological approaches. *J. Natl Cancer Inst.*, **81,** 1680–1681.